# Decoding complex biological networks - tracing essential and modulatory parameters in complex and simplified models of the cell cycle

**DOI:** 10.1186/1752-0509-5-123

**Published:** 2011-08-07

**Authors:** Olivia Eriksson, Tom Andersson, Yishao Zhou, Jesper Tegnér

**Affiliations:** 1 Division of Mathematical Statistics, Department of Mathematics, Stockholm University, SE-106 91 Stockholm, Sweden; 2 Division of Mathematics, Department of Mathematics, Stockholm University, SE-106 91 Stockholm, Sweden; 3 Unit for Computational Medicine, Department of Medicine, Center for Molecular Medicine, Karolinska Institutet, Karolinska University Hospital, Solna, SE-171 76 Stockholm, Sweden

## Abstract

**Background:**

One of the most well described cellular processes is the cell cycle, governing cell division. Mathematical models of this gene-protein network are therefore a good test case for assessing to what extent we can dissect the relationship between model parameters and system dynamics. Here we combine two strategies to enable an exploration of parameter space in relation to model output. A simplified, piecewise linear approximation of the original model is combined with a sensitivity analysis of the same system, to obtain and validate analytical expressions describing the dynamical role of different model parameters.

**Results:**

We considered two different output responses to parameter perturbations. One was qualitative and described whether the system was still working, i.e. whether there were oscillations. We call parameters that correspond to such qualitative change in system response *essential*. The other response pattern was quantitative and measured changes in cell size, corresponding to perturbations of *modulatory *parameters. Analytical predictions from the simplified model concerning the impact of different parameters were compared to a sensitivity analysis of the original model, thus evaluating the predictions from the simplified model. The comparison showed that the predictions on essential and modulatory parameters were satisfactory for small perturbations, but more discrepancies were seen for larger perturbations. Furthermore, for this particular cell cycle model, we found that most parameters were either essential or modulatory. Essential parameters required large perturbations for identification, whereas modulatory parameters were more easily identified with small perturbations. Finally, we used the simplified model to make predictions on critical combinations of parameter perturbations.

**Conclusions:**

The parameter characterizations of the simplified model are in large consistent with the original model and the simplified model can give predictions on critical combinations of parameter perturbations. We believe that the distinction between essential and modulatory perturbation responses will be of use for sensitivity analysis, and in discussions of robustness and during the model simplification process.

## Background

In recent years, numerous studies characterizing static topological properties of molecular networks derived from heterogeneous sources such as metabolic, transcriptomic, transcription factor and protein-protein interaction data have been published [1]. Concurrently, several studies have revealed the dynamics of small-scale molecular networks using innovative applications of reverse-engineering techniques, modeling and computer simulation of kinetic equations [2]. However, observations regarding overall parameter robustness of biological circuits may imply that beyond the apparent model complexity there exists, in a mathematical sense, one or several core systems or operational principles driving the system dynamics that are shielded by the more complex original dynamical equations [3-6]. Such a dynamical core is not necessarily apparent from the connectivity graph or from the original dynamical equations. The existence of such a core has been supported by studies employing an ensemble simulation approach in the analysis of various computational models [7,8]. Essentially, a large number of different parameter configurations exist that produce the "same" dynamical output from the system, as emphasized in a study by Gutenkunst et al [9], whereby, using a large set of biochemical models, they found a "sloppy" parameter sensitivity spectra to be a universal characteristic of these models.

To advance our understanding of molecular networks, we need to develop methods that permit an analysis of such underlying core dynamics. Research on model reduction and reformulation has been at the forefront within the systems biology community over the last few years (e.g. [4,10-16]). Several studies have targeted the elaborate original model using different approaches, and have arrived at a more compact system description, which in different ways capture some aspects of the essence of the original model dynamics. Using *model reduction *tools, the number of degrees of freedom are reduced, while still remaining within the same model formalism as the original model. By *reformulating the model*, the original model is approximated to another hopefully more transparent modeling formalism, with a structure that better captures the key qualities of the original system. Examples of model reduction [17] include lumping of variables [10,11], separation of timescales [14] (or, for example, the classical Michaelis-Menten equation describing enzyme kinetics), sensitivity analysis based methods, and methods based on identifiability analysis [13]. Examples of transformation of modeling formalism include boolean approximations [4,12], hybrid stochastic approximations [18], or the simplified model used throughout this study, a delayed piecewise linear approximation [15] of an ordinary differential model.

**Figure 1:**
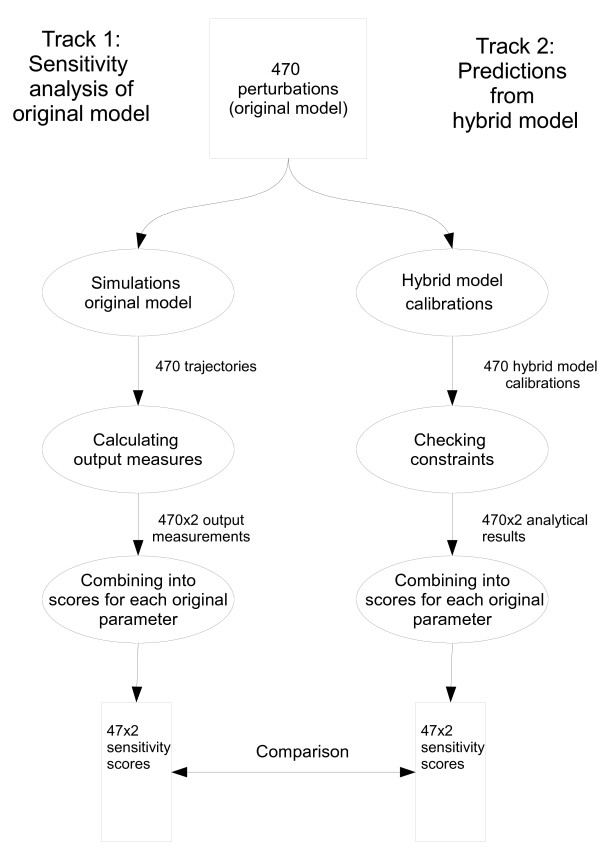
**Work flow**. We compared a sensitivity analysis of the original model (Track 1) to predictions from the hybrid model (Track 2) using the same set of 470 perturbations of original model parameters. Track 1: For each perturbation i) a numerical simulation was performed; ii) output measures were retrieved (cycle time *t_CT _*and cell mass *M_end_*) and; iii) sensitivity scores ( and ) were constructed for each parameter. Track 2: For each perturbation of the original model i) a corresponding recalibration of the hybrid model parameters was performed; ii) the analytical constraints (NC and SF) were recalculated and; iii) sensitivity scores ( and ) were constructed for each *original *parameter.

The simplified model has subsequently been used to characterize parameters of the original model, as in Radulescu et al [14], where critical parameters are found through reduction to dominant subsystems, and after this analytically mapped back to the original model. Here, in contrast, we started from the original model, by exploring the dynamics with sensitivity analysis, and next compared this to the predictions from the simplified model. The aim was to investigate to what extent it is feasible, by using an underlying core description [15], to explain dynamical properties of the original model (Figure 1).

The cell cycle is one of the most extensively studied and essential biological regulatory circuits. The differential computational models pioneered and developed by Novak and Tyson [2,19] describing cell cycle regulation of fission yeast, incorporates and accounts for a large body of experimental data. During the cell cycle, the cell grows, DNA replicates, and the cell divides into two daughter cells. To maintain cell size over several generations, it is essential that there is a controlled balance between cell growth and cell division. The model used in the present study [19] describes the key components of that regulatory mechanism, consisting of proteins and protein complexes that drive the cell cycle in fission yeast. It is detailed in the Methods (equations (3-16)) and is referred to throughout as the NT-model. The NT-model is thus our point of departure for the model simplification process and sensitivity analyses. For a detailed description of the NT-model see [19]. For a more general discussion of cell cycle dynamics see [2].

To examine the dynamical properties of the original NT-model, we performed a sensitivity analysis. Two different types of output responses to parameter perturbations were considered. One was qualitative and described whether the system was still working, i.e. whether there were oscillations (cell divisions) corresponding to parameters that here we call "essential". The other response pattern was quantitative and measured changes in cell size (cell growth), which described "modulatory" effects of parameter perturbations. This is primarily a methodological distinction, i.e. response effects related to parameter perturbations. In principle, parameters could be both essential and modulatory, depending on the perturbations in question. In practice, when conducting sensitivity analyses, the key question is which parameters affect the system output, how, and under what conditions. Here, we extend the scope of the sensitivity analysis to evaluate the correspondence between perturbation effects in the original model and analytical results of the simplified model, focusing on two types of possible perturbation effects - essential and modulatory changes - anticipating that the simplified model should be able to account for both of these situations. The analysis includes three key steps: (1) perturbations of the parameters of the original NT-model; (2) a corresponding recalibration of the parameters of the simplified model; and (3) systematic comparison of the perturbation effects on the orginal and simplified models (Figure 1). The perturbation effects of the original model are investigated by numerical simulations whereas the corresponding effects of the simplified model are retrieved by evaluation of analytical expressions.

**Figure 2:**
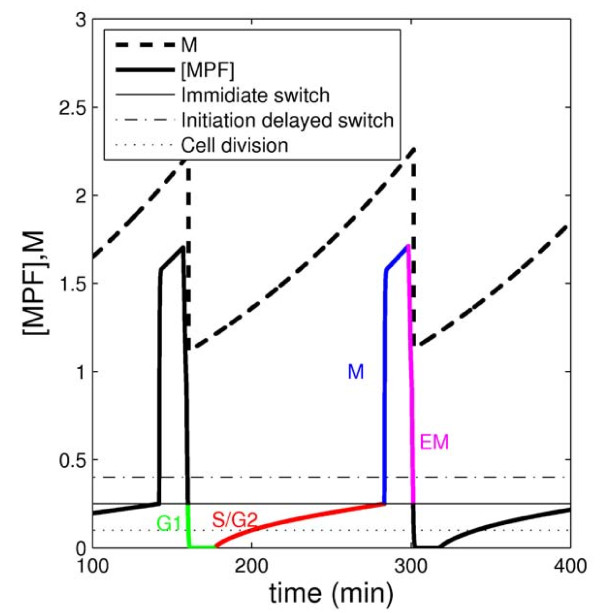
**Cell cycle dynamics**. Numerical simulation of the hybrid, DPL-model with the different linear systems indicated. During different parts of the cell cycle trajectory, different linear systems are used, here indicated on the time course of the variable *y*(*t*) = [*MPF*](*t*) with green, red, blue and magenta. These linear systems correspond to the four cell cycle phases G1, S/G2, M and EM, where EM is the ending of Mitosis. Mathematically, the linear system used at time *t *depends on [*MPF*](*t*) and *t' *(the time since the last occasion when [*MPF*] = *θ*_*slp*/*ste *_as detailed in [15]), and the linear systems are, green: [*MPF*] <*θ*_25/*wee *_and *t' *<*τ *(the system matrix **A**_12 _is used), red: [*MPF*] <*θ*_25/*wee *_and *t' *>*τ *(**A**_11_), blue: [*MPF*] >*θ*_25/*wee *_and *t' *<*τ *(**A**_21_), and magenta: [*MPF*] >*θ*_25/*wee *_and *t' *>*τ *(**A**_22_). The default parameter set of [15] was used in this figure.

In [15] we introduced a scheme by which we could simplify the NT-model using step functions and a time delay. Introducing discrete step-functions resulted in a hybrid model containing both continuous and discrete variables. In the case of the NT-model, this hybrid model turned out to be piecewise linear. This *delayed piecewise linear (DPL)*-model can be found in the Methods (equations (17-20)), and is referred to throughout as the hybrid, or the DPL-model. A piecewise linear description facilitates system analysis, as each individual linear system can be analyzed separately using well established linear system analysis tools. The combined results from these separate analyses exposes the behaviour of the full system (Figure 2).

Sensitivity analysis refers to a broad range of mathematical and statistical analysis methods that serve to evaluate the relative merits of a set of parameters in controlling system dynamics. The aim is to relate variation (uncertainty) in the system output to variation (uncertainty) in the system input. The analysis method chosen depends on the goal. Campolongo et al [20] distinguish between three types of analysis: (1) factor screening; (2) local sensitivity analysis; and (3) global analysis. The first type refers to an analysis targeting the effects of parameter variation one at a time, i.e. variation in system output is related to parameters one at a time. It is a more or less qualitative assessment of the relevant factors in system dynamics. By contrast, local and global sensitivity analyses imply detailed quantitative assessments of relative effects of the isolated factors of concern. Local analysis makes use of differential calculus, and stability analysis of equilibrium points. Global sensitivity analysis targets response effects in the whole parameter space, as well as interaction effects between parameters. It relies to a larger extent on statistical methods. Our purpose in this study was to clarify the relevance of parameters in cell cycle dynamics. As a starting point, we performed a screening study to investigate the relative merits of single parameters in cell cycle regulation.

## Results

Our analysis is based on a set, , of parameter perturbations of the original NT-model, consisting of 470 single parameter changes. This set was used to analyse the relationship between parameter values and model output (cell size and cycle time) through two different tracks, see Figure 1. During the first track, the sensitivity analysis, numerical simulations of the NT-model were conducted for all perturbations in , and scores of sensitivity were constructed for each model parameter. During track two, all perturbations of  were translated to corresponding perturbations of the hybrid model and, instead of simulations, mathematical conditions were evaluated in order to predict the sensitivity to the perturbations, and thereby construct scores of sensitivity. Two measures of sensitivity were used in both tracks, targeting *essential *and *modulatory *effects of perturbations, respectively. Finally, the sensitivity scores from the two tracks were compared in order to i) validate the predictions from the hybrid model, and ii) investigate the role of essential and modulatory parameters and perturbations. The results are divided into the following sections: (I) sensitivity analysis of original model; (II) analytical predictions from the hybrid model; and (III) comparison between the analytical predictions and the sensitivity analysis. Finally, (IV) the hybrid model is used for more complicated predictions.

### I Sensitivity analysis of the original model identifying essential and modulatory perturbation effects

The parameters of the original model, *k*1,..., *μ *= **p***^org ^*(equation (16)), were perturbed one at a time, starting from a default set of parameter values corresponding to the wild-type cell (equation (16)). A perturbation consists of a change of the parameter value by a factor *ps_j _*(the relative Perturbation Size), where *ps_j _*∈ **ps **= (10^-1^, 10^-0.8^, 10^-0.6^, 10^-0.4^, 10^-0.2^, 10^0^, 10^0.2^, 10^0.4^, 10^0.6^, 10^0.8^, 10^1^). Each of the 47 parameters, , *i *∈ 1,..., 47, was thus perturbed 11 times given the new value , *j *∈ 1,..., 11, where  is the default value of parameter . For each perturbed simulation, the output trajectory was examined for i) whether there were consistent oscillations or not, ii) cycle time *t*_*CT *_and iii) final (before cell division) cell mass *M*_*end*_.

**Figure 3:**
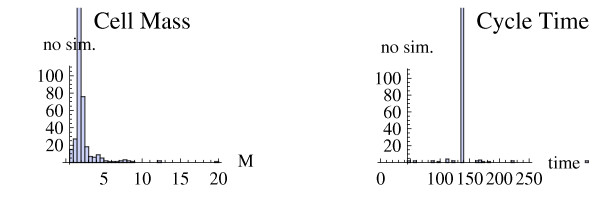
**Different distributions of cell mass and cycle time**. Histograms of cell mass (*M_end_*) and cell cycle time (*t_CT_*) respectively. Cell mass was measured at the end of the last cell cycle of each simulated trajectory, just before cell division and was 2.0 for the default parameter set. Only trajectories with proper oscillations (defined in the main text) were used. Cycle times for the two last cycles of each trajectory are plotted. The cycle time of the default parameter set was 138.6.

i) Out of a total of 470 perturbations (47 × 10, not counting *ps_j _*= 1), 437 corresponded to trajectories with consistent oscillations. ii) When inspecting these trajectories, two distinct patterns emerged - either the cycle time *t_CT _*was constant within the trajectory, or, for a few of the perturbations, *t_CT _*alternated in a repeated pattern, e g. *t_CT _*= ..., 111.0, 166.25, 111.0, 166.25, 111.0,... (denoted *quantized *cell cycles using the formulation of Novak and Tyson [19]). Surprisingly, for perturbations with constant cycle times, most had the same cycle time *t_CT _*= 138.6 (in total 423 perturbations), giving a very narrow distribution of cycle times (see Figure 3). One exception to this was the parameter *μ*, which determines the speed of cell growth, and naturally has a large impact on cycle time. iii) For final cell mass *M_end_*, the pattern reversed. The parameter perturbations generated a broader distribution of *M_end _*(Figure 3).

We next defined measures of sensitivity summarizing how sensitive the system was to perturbations of the single parameter . Two measures were defined, one based on the cycle time of the oscillations, denoted , and the other based on final cell mass, . Since the cycle time was, in almost all cases, either constant *t_CT _*= 138.6 or non-existing (quantized cycle times or no oscillations), we decided to consider only whether we had proper cell cycle oscillations or not in the definition of . Proper oscillations were defined as trajectories that still oscillated after 4000 min, and had a narrow distribution of cycle times (to remove quantized behaviour). We therefore ended up with one discrete-qualitative measure  and one continuous-quantitative . In the calculation of , only simulations with proper oscillations were considered. We denoted perturbations with a large effect on *S^osc ^*essential, and with a large effect on  modulatory.

The sensitivity analysis was carried out using two different ranges of parameter perturbations, *narrow range *perturbations close to the default parameter value, and *wide range *perturbations both close, but also further away. For narrow range perturbations,  and  were calculated using only the two perturbations closest to the default value, i.e. **ps***_narrow _*= (10^-0.2^, 10^0^, 10^0.2^), while for wide range, all perturbation sizes were used, **ps***_wide _*= **ps**.

#### Essential parameters

The sensitivity measure, , corresponds to the number of perturbed simulations of parameter  that ended in cell collapse, i.e. a lack of proper oscillation, , where *s_ij _*= 0 if perturbation *j *resulted in proper oscillations, and *s_ij _*= 1 otherwise, *j *∈ 1,..., *n_pert_*, where *n_pert _*= 11 for wide and *n_pert _*= 3 for narrow range perturbations. For narrow range perturbations, all parameters have  (Table 1 original model), and therefore the system is robust against perturbations of this size when proper oscillations are considered. We here define robustness as persistent system dynamics after perturbation. It is quantified with the sensitivity scores (being the opposite to sensitivity). Even for larger perturbations, oscillation is a robust behaviour for most parameter perturbations (Table 1 original model, wide range perturbations). Still, some perturbations do affect oscillations, e.g. the rate constants *k*_9 _and *k*_10 _of the hypothesized intermediate enzyme IEP, of the Slp1/APC subsystem, which remove the protein complex MPF at the end of mitosis. In the coming analysis, we denote parameters with  as essential, and with  as non-essential.

**Table 1:** Essential perturbation effects - comparison between the hybrid model predictions and the original model results

Essential Parameter Effects					
**Narrow Range**			**Wide Range**		
	
**Parameter**	**Original**	**Hybrid**	**Parameter**	**Original**	**Hybrid**

	0	0	*k*_10_	**6**	**1**
	0	0	*k*_9_	**5**	**1**
	0	0	*k*_6_	**4**	**4**
	0	0		**4**	**4**
*J*_*i*25_	0	0	*k*_14_	**3**	0 *
*J*_*a*25_	0	0	*k*_13_	**3**	0 *
*V*_*i*25_	0	0	*k*_8_	**3**	**3**
*V*_*a*25_	0	0	*k*_7_	**3**	**4**
*J_iwee_*	0	0	*J*_5_	**3**	**4**
*J_awee_*	0	0		**2**	0
*V_iwee_*	0	0		**1**	**1**
*V_awee_*	0	0	*J*_7_	**1**	0
*J*_16_	0	0 *		0	0
*J*_15_	0	0 *		0	0
	0	0 *		0	0
	0	0 *	*J*_*i*25_	0	0
*k*_15_	0	0 *	*J*_*a*25_	0	0
*k*_14_	0	0 *	*V*_*i*25_	0	0
*k*_13_	0	0 *	*V*_*a*25_	0	0
*k_diss_*	0	0 *	*J_iwee_*	0	0
	0	0 *	*J_awee_*	0	0
	0	0 *	*V_iwee_*	0	0
*k*_12_	0	0 *	*V_awee_*	0	0
*k*_11_	0	0 *	*J*_16_	0	0 *
*J*_10_	0	0	*J*_15_	0	0 *
*J*_9_	0	0		0	0 *
*k*_10_	0	0		0	0 *
*k*_9_	0	0	*k*_15_	0	0 *
*J*_8_	0	0	*k_diss_*	0	0 *
*J*_7_	0	0		0	0 *
*k*_8_	0	0		0	0 *
*k*_7_	0	0	*k*_12_	0	0 *
*J*_5_	0	0	*k*_11_	0	0 *
*k*_6_	0	0	*J*_10_	0	0
	0	0	*J*_9_	0	0
	0	0	*J*_8_	0	0
*J*_4_	0	0		0	0
*k*_4_	0	0	*J*_4_	0	0
	0	0	*k*_4_	0	0
*J*_3_	0	0		0	0
	0	0	*J*_3_	0	0
	0	0		0	0
	0	0		0	0
	0	0		0	0
	0	0		0	0
*k*_1_	0	0	*k*_1_	0	0

Comparison between the sensitivity analysis of the original model and the analytical predictions of the hybrid model (mapped back to the original model parameters), at small range and wide range parameter perturbations. The sensitivity scores  (original model) and  (hybrid model), describing essential perturbation effects are used. The parameters are sorted by the sensitivity scores (based on the original model) in decreasing order; * indicates original model parameters without a counterpart in the hybrid model. Scores corresponding to essential effects (, ) are in bold.

#### Modulatory parameters

**Figure 4:**
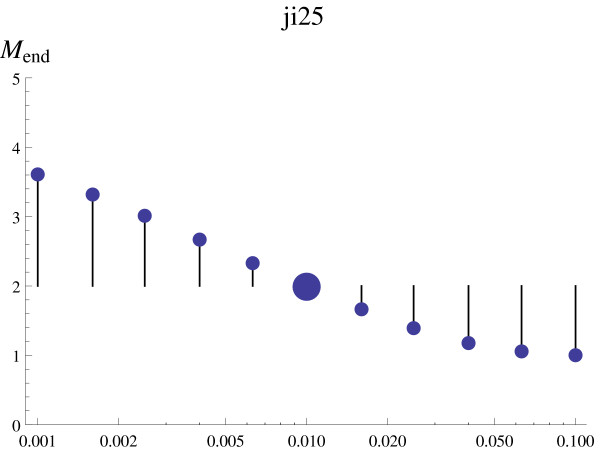
**Calculation of the ***S^mass ^***score**. The distance between the perturbed final cell mass,  (small dots) and the default final cell mass  (large dot), for each perturbation of parameter *J*_*i*25_. The sensitivity score  is calculated by the average distance of all perturbations.

The sensitivity measure, , corresponds to the average effect on final cell mass *M^end ^*of the *n *perturbations of parameter  that had proper oscillations (*s_ij _*= 0) (Figure 4). It is defined as , where *j*' corresponds to perturbations with *s_ij _*= 0,  is the final cell mass after the *j*'th perturbation of parameter , and  is the final cell mass using the default parameter set (16).

With narrow range perturbations (Table 2 original model) the parameters with the highest sensitivity  are related to either the proteins Cdc25 or Wee1, or to the formation or destruction of the protein Cdc13. This is in agreement with the fact that, in the original NT-model, final cell mass is in large determined by the SNIC bifurcation [19] corresponding to the G2/M checkpoint. This checkpoint is realized by the phosphorylation and dephosphorylation of Cdc25 and Wee1.

**Table 2:** Modulatory perturbation effects - comparison between the hybrid model predictions and the original model results

Modulatory Parameter Effects					
**Narrow Range**			**Wide Range**		

**Parameter**	**Original**	**Hybrid**	**Parameter**	**Original**	**Hybrid**
	
*k*_1_	**0.637**	**0.593**	*k*_1_	**3.98**	**3.84**
	**0.533**	**0.507**		**2.35**	**2.17**
*V*_*i*25_	**0.523**	**0.25**		**1.77**	**2.35**
*V*_*a*25_	**0.521**	**0.249**	*V*_*i*25_	**1.27**	**0.764**
	**0.361**	**0.502**	*V*_*a*25_	**1.27**	**0.762**
	**0.225**	**0.31**	*k*_15_	**0.966**	0. *
*J*_*i*25_	**0.222**	**0.29**		**0.965**	0. *
	**0.168**	**0.106**		**0.948**	0.0102
	0.0367	0.0165		**0.859**	**0.259**
*V_iwee_*	0.0356	**0.237**	*k*_13_	**0.83**	0. *
*k*_13_	0.0352	0. *	*k*_14_	**0.823**	0. *
*k*_14_	0.0345	0. *		**0.801**	**1.08**
*V_awee_*	0.0343	**0.234**	*J*_*i*25_	**0.786**	**1.01**
*k*_11_	0.0307	0. *		**0.555**	**0.408**
*J*_4_	0.0259	0.0148	*V_iwee_*	**0.304**	**0.65**
	0.0237	0.0148	*V_awee_*	**0.303**	**0.649**
	0.0212	0. *	*k*_9_	**0.207**	**0.396**
*k*_9_	0.0212	0.0191	*k*_11_	**0.202**	0. *
	0.02	1.64 × 10^-7^	*J*_4_	**0.157**	0.0941
*k*_7_	0.0181	0.000916		**0.149**	0.094
	0.0142	0.000213		0.0817	0. *
	0.0142	0.000237		0.065	0.00137
	0.0135	0.00256	*k*_7_	0.0491	0.00615
*k*_6_	0.0118	0.000217		0.0421	0.000484
	0.00821	0. *	*J_awee_*	0.0392	0.097
*J_awee_*	0.00655	0.017	*J*_*a*25_	0.0366	0.0435
*k*_4_	0.00624	0.0134	*k*_4_	0.0354	0.0632
*J*_*a*25_	0.00574	0.00586		0.029	0. *
*k*_8_	0.00574	0.000655		0.0238	0. *
*k*_10_	0.00548	0.0189	*k*_10_	0.022	**0.396**
*J*_5_	0.00291	0.000132	*k*_8_	0.0127	0.00918
*k*_15_	0.00282	0. *		0.0102	3.04 × 10^-7^
	0.00232	0. *	*J*_5_	0.00917	0.0000567
	0.000926	0. *	*k*_6_	0.00828	0.000456
	0.000595	0.00173		0.00567	0.0331
	0.000429	5.63 × 10^-9^	*J*_15_	0.00219	0. *
*J*_15_	0.000331	0. *		0.00189	3.15 × 10^-8^
*k*_12_	0.000263	0. *	*k*_12_	0.00144	0. *
*J_iwee_*	0.000166	0.00304	*J*_3_	0.000874	0.000998
*J*_10_	0.000166	0.0007	*J*_9_	0.000724	0.00169
*J*_8_	0.000166	0.000233	*J_iwee_*	0.000723	0.00895
*J*_3_	0.000166	0.00016	*J*_10_	0.000694	0.00437
*J*_16_	0.	0. *	*J*_8_	0.000306	0.00149
*k_diss_*	0.	0. *	*J*_16_	0.000271	0. *
*J*_9_	0.	0.00027	*k_diss_*	0.000207	0. *
*J*_7_	0.	1.72 × 10^-7^	*J*_7_	0.0000497	1.1 × 10^-6^

Comparison between the sensitivity analysis of the original model and the analytical predictions of the hybrid model (mapped back to the original model parameters), at small range and wide range parameter perturbations. The sensitivity scores  (original model) and  (hybrid model), describing modulatory perturbation effects are used. The parameters are sorted by the sensitivity scores (based on the original model) in decreasing order; * indicates original model parameters without a counterpart in the hybrid model. Scores corresponding to modulatory effects (, ) are in bold.

With wide range perturbations (Table 2 original model), the top scoring parameters also include  and *k*_15_, which are related to TF (a transcription factor) activity, and  related to the activity of the protein Ste9. This is most likely a result of the G1/S checkpoint not functioning properly in the presence of these large parameter perturbations further away from the default parameter set, and being active at a point where it should not. In the coming analysis, we denote parameters with  as modulatory and with  as non-modulatory. This, a bit arbitrarily chosen, limit  corresponds to a relative average change of cell size of 5%.

### IIA Analytical predictions from the hybrid model on essential and modulatory perturbation effects

In [15] we introduced a scheme by which we could simplify the NT-model. Dynamical switching modules were identified and replaced by time lagged step-functions, resulting in the hybrid DPL-model. This model approximation process is summarized in the Methods. The hybrid model (equations (17-19)) consists of four linear systems, defined by the matrices **A***_kl_*, *k*, *l *∈ {1, 2}, where the linear system at each time-point *t *is determined by, so called, switching rules (equation (18)). Figure 2 displays a numerical simulation of the DPL-system, indicating the different linear systems. A switch between systems is initiated when the output *y*(*t*) = [MPF](*t*), corresponding to the concentration of the protein complex MPF, crosses a threshold *θ*_25/*wee *_or *θ*_*slp/ste*_. Note that when *θ*_25/*wee *_is passed, the switch is immediate, whereas, when *θ*_*slp/ste *_is passed, the switch is delayed with *τ *minutes. The switching rule can therefore be considered as equipped with a memory, and we have denoted the model *delayed *piecewise linear. An analysis of the DPL-model [15] showed that each separate linear system, defined by the system matrix **A***_kl_*, was stable, having an asymptotically stable fixed point (, with real negative eigenvalues). Combining the four linear systems with the switching rules revealed, however, that some of the fixed points could never be reached without passing a switching threshold (and thereby a change of linear systems). Thus, these fixed points were no longer stable. The relationship between the location of the fixed points and the switching thresholds, therefore, determines the dynamical behaviour of the system. This relationship between fixed point and switching thresholds corresponds to analytical expressions including the parameters of the hybrid model, and allows us to present two types of important parameter/output relationships. The first type refers to necessary constraints (NC), which must be satisfied in order for the model to generate proper oscillations. Therefore NC identifies essential parameter changes. The second type is a function describing the size of the cell, or Size Function (SF). SF describes how cell size is influenced by hybrid model parameters, and therefore SF identifies modulatory parameter changes and their effects on cell size. When using these expressions in order to find out whether a parameter change is modulatory, essential or both, we always have the default parameter set as the point of departure for the change. With another set of default parameter values, the result could of course change. Note that NC is a qualitative test - either a parameter change violates the conditions (and the change is considered to be essential), or it does not; whereas SF is quantitative, giving numerical predictions on cell size.

#### Analytical results identifying essential parameter changes - necessary constraints NC

Two constraints, that are necessary in order to have cell cycle oscillations, were retrieved in the analysis of the hybrid model [15], and are further extended in the Methods section (equations (24,25)). These correspond to inequalities, including hybrid model parameters, that define regions of parameter values for which there cannot be proper oscillations (note that the necessary conditions only state that if not satisfied, there will not be oscillations, but not the opposite, i.e. they do not assure oscillations, which agrees with the definition of "essential" in the sensitivity analysis, i.e. anhilation of oscillation due to parameter perturbations). A change of hybrid parameter *τ*...*μ *= **p***^hyb ^*(equation (20)), that violates these constraints is considered as essential.

#### Analytical results identifying modulatory parameter changes - the size function SF

The second type of parameter/output relationship, the function SF (equations (21,22), Methods), describes how the final size of the cell (i.e. just before cell division), is influenced by hybrid model parameters. SF corresponds to the formation of a concentration threshold. When the concentration of the protein complex MPF passes this threshold (under normal circumstances), cell division is initiated. In cell cycle physiology, it corresponds to the G2/M checkpoint [2].

### IIB Translating the analytical results from the hybrid model to the original model

To be able to compare the hybrid and original models (i.e. compare their respective parameter characterizations) we needed to translate the analytical result of the hybrid model to the original model. For this translation, we first defined a mapping from original model parameters to hybrid model parameters. We then used this mapping to backtrack the analytical results of the hybrid model to the original model parameters.

#### Mapping original model parameters onto hybrid model parameters

There is no simple algebraic relationship that could be used to define mappings between original and hybrid model parameters parameters, , *i *= 1...*n_org _*and , *m *= 1...*n_hyb_*, i.e. there is no analytical function  for all *m *that can be used to relate the original and hybrid models. Instead, we constructed mappings numerically for different specific values of **p***^org^*, namely those values corresponding to the perturbations used in the sensitivity analysis. First, each original parameter  was perturbed *n_pert _*= 11 times according to the relative perturbation sizes, **ps**, described earlier, i.e. the parameter was given the value , *j *= 1...*n_pert _*(note here that single index  denotes a parameter, whereas a double index  denotes a specific parameter value). Next, the effect from the perturbation was translated to the hybrid parameters **p***^hyb^*. The translation was done using a recalibration process, adapting the hybrid model to the original perturbed one through re-performing the hybrid approximation process and measuring the effect of the perturbation  on  for all *m *= 1...*n_hyb _*(Methods). This means that we, for each perturbation of the original model , *j *= 1...*n_pert_*, *i *= 1,..., *n_org_*, obtained a new set of hybrid mobel parameter values, denoted by .

#### Backtracking the analytical result from the hybrid model to the original model

After recalibration of the hybrid model, we translated the analytical result in terms of modulatory and essential effects, by backtracking the analytical conditions (NC and SF) to the original model. This means that for each perturbation of an original model parameter , we constructed a new hybrid model and examined the analytical expressions determining essential and modulatory effects. From this, we finally characterized the original parameter in terms of essential and/or modulatory. To sum up, the essential and modulatory effects of the original parameter perturbations are measured in two main ways: with respect to the original model (track 1), and with respect to analytical constraints and implications after recalibration of the hybrid model (track 2).

##### Bactracking the essential conditions

To backtrack the necessary constraints (NC, corresponding to essential parameter changes), to original parameter characterizations, we counted the total number (out of *n_pert_*) of perturbations of  which have a mapped effect such that the necessary constraints (equations (24,25)) are violated. From this we calculated a sensitivity score similar to the one from the sensitivity analysis, but based on the hybrid model, and therefore denoted . Thus, , where  if perturbation *j *of parameter  (mapped to the hybrid model) does not violate constraints (24,25) and  otherwise, *j *∈ 1,..., *n_pert_*.

##### Backtracking the modulatory conditions

To backtrack the function determining cell size (SF, corresponding to modulatory parameter changes) to original parameter characterizations, we calculated the average mapped effect that the *n *perturbations of  (which do not violate the necessary constraints (24,25)) should have on final cell size, as calculated by SF. By this we get a calculated sensitivity score similar to the one from the sensitivity analysis, but based on the hybrid model and therefore denoted . Thus, , where  is the, from SF (equations (21,22)), calculated final cell size after the *j*'th perturbation of parameter  (mapped to the hybrid model), where *j*' corresponds to those *j *for which , and  is the by SF calculated final cell size using the default parameter set (20).

There are two hybrid parameters that are not considered in the correspondence analysis. The first one is *μ*, which is related to the growth of the cell, i.e. the increase in cell mass *M*. Since the hybrid model is based on the assumption that the increase in *M *is very slow compared to other variables, perturbations of *μ *are not considered. The other parameter is *τ*, which has not been included in the analysis for practical reasons (Methods). Not all original parameter perturbations have a mapping to a hybrid parameter (data not shown). This is partly a reflection of the fact that parts of the original system are inactive during the experimental context that the hybrid model seeks to reproduce, and has therefore not been included in the hybrid model.

### III Correspondence analysis of hybrid and original parameter characterizations

Once we had conducted the sensitivity analysis of the original model (I), and retrieved the corresponding predictions from the hybrid model (II), we were now in a position to compare the results (Figure 1). The parameters were divided into essential or non-essential (Table 1) and modulatory or non-modulatory (Table 2), with respect to the model in question: original or hybrid. We considered both narrow range and wide range perturbations. A parameter was characterized as modulatory if  (or ) and as essential if  (or ), and the parameter characterizations of the original and hybrid models were compared.

No parameter was detected as being essential by narrow range perturbations, neither from the original nor hybrid model (Table 1). Wide range perturbations were needed to get an essential effect. For modulatory characterizations, the correspondence was better for the models at narrow range perturbations than at long range perturbations (Table 2). The result is summarized by a binary (essential/non-essential or modulatory/non-modulatory) classification test (Table 3). We view the parameter characterizations from the hybrid model as a predictor of the class of the original model parameters, and calculate the sensitivity and specificity of the predictions for each class (Table 3). For the narrow range modulatory perturbations, the prediction is close to perfect, with a sensitivity of 100% and a specificity of 95% (Table 3). For narrow range essential predictions the specificity is 100% and no hybrid or original model parameters are found to be essential. When wide range perturbations are considered, there are more mis-classifications. For modulatory parameter predictions the sensitivity decreased to 60%, while the specificity was 96% and for essential predictions, the sensitivity was 67% and the specificity remained 100% (Table 3).

**Table 3:** Sensitivity and specificity of hybrid model predictions

Prediction	sensitivity	specificity
modulatory-narrow range	100%	95%
modulatory-wide range	60%	96%
essential-narrow range	-	100%
essential-wide range	67%	100%

Note that for narrow range no perturbations are found to be essential, neither by the original or hybrid model.

In the hybrid model, modulatory parameters are defined by the size function SF, which corresponds, as earlier described, to the G2/M checkpoint of the cell cycle. The proteins behind this checkpoint are those that are most important to regulate the size of the cell in this context. If we did not know this and were to identify the mechanism regulating cell size, from this model comparison, we can see that we would be better of by the use of narrow perturbations rather than wide ones. If wide range perturbations are used, other parameters, which are not involved in this checkpoint, are also incorrectly identified, and therefore confusing the picture.

We can conclude that the correspondence between models is better near the default values of the model parameters (narrow range), and becomes worse further from this point. At the same time, it seems impossible to identify essential parameters without using perturbations further away from the default parameter setting. Finally, if we use narrow range perturbations to identify modulatory parameters and wide range perturbations to identify essential parameters, then we note that most parameters are either essential or modulatory for this set of perturbations.

#### Mis-predictions

There are two main types of mis-predictions at wide range perturbations. First, parts of the original model, for which there is no counterpart in the hybrid model (parameters indicated with * in Table 1 and Table 2), and that are not active at narrow range perturbations, have in fact an essential or modulatory effect at wide range perturbations. This indicates that the hybrid model performs quite well for predictions locally around the point in parameter space where the hybrid model was constructed. Further away from this point, the predictions are still quite good for those parts of the original system that were included in the hybrid model. The hybrid model cannot, however, capture what will happen to parts that were not included. Second, there are some mis-predictions that are most likely due to the approximation and discrepancy between the hybrid and original models. One such prediction was the parameters *k*_9 _and *k*_10_, which are found to be more essential in the original model than in the hybrid model. *k*_9 _and *k*_10 _are important actors to obtain quantized output behaviour. Such behaviour cannot be mimicked by the hybrid model; rather, the hybrid model predicts that the system will have proper oscillations for some of the perturbations, which gives quantized oscillations. Also, the fact that we have not included the hybrid model parameter *τ *in the analysis might affect predictions.

Finally, we have a problem with mappings to the hybrid model parameter *θ*_25/*wee*_. This parameter is different compared to the other hybrid parameters since it corresponds to the switching threshold of *two *step functions, *s*_25 _and *s_wee _*(Methods), when their respective switching thresholds are at the same level i.e. *θ*_25/*wee *_= *θ*_25 _= *θ_wee _*when *θ*_25 _= *θ_wee_*. To be able to map a perturbation of the original model to *θ*_25/*wee *_∈ **p***^hyb ^*that keeps this relationship (*θ*_25 _= *θ_wee_*), and thus the structure of the hybrid model, at least two original parameters have to be perturbed simultaneously. However, the *single *parameter perturbations  used in this study, only correspond to a change in either *θ*_25 _or *θ_wee_*. To obtain a numerical value for each mapping  despite this, we assume that *θ*_25/*wee *_= min(*θ*_25_,*θ_wee_*), when the threshold is passed from below, and that *θ*_25/*wee *_= max(*θ*_25_, *θ_wee_*), when the threshold is passed from above. Presuming that as soon as either *θ*_25 _or *θ_wee _*is passed, the other threshold will also soon be passed.

### IV Combinatorial predictions from the hybrid model

After investigating the correspondance between the original and hybrid model concerning *single *parameter perturbations of the original model, we used the hybrid model to identify *combinations *of parameters aimed at a specific critical effect.

#### Essential combinations of parameters

We used the necessary constraints (24,25) to find *hybrid *model parameters with an essential effect on model output. To evaluate the constraints, we used, as earlier, a set of perturbations, but this time investigating hybrid model parameters directly instead of translating the result to original model parameters. From these perturbations (Table 4) we could see that (at least) hybrid model parameters *θ*_25/*wee*_, *h*_*wee *_and *h*_*slp*/*ste *_can have an essential effect.

**Table 4:** Essential hybrid model parameters

Parameter (p*^hyb^*)	**no**.
*k*_1_	0
	0
*l*_25_	0
*h*_25_	0
*θ*_25/*wee*_	2
*h_wee_*	1
*l_wee_*	0
*l*_*slp*/*ste*_	0
*h*_*slp*/*ste*_	1
*θ*_*slp*/*ste*_	0

The hybrid model parameters were perturbed one by one with the relative perturbation sizes *ps_j _*∈ **ps **(as defined in Results), and the constraints (24) and (25) were evaluated. The table shows the total number of perturbations (out of 11) for which the constraints were violated.

The hybrid parameter *θ*_25/*wee *_was shown to have the largest effect (Table 4), using these perturbations. Simulataneously, none of the original parameters mapped to *θ*_25 _or *θ_wee _*were found to be essential (data not shown). *This emphasizes that there are important (essential) features in the original model that are robust towards single parameter perturbations*. We described earlier that *θ*_25/*wee *_∈ **p***^hyb ^*can only be mapped to from combinations of original parameters if the structure of the hybrid model is to be retained (i.e. *θ*_25 _= *θ_wee_*). We therefore expect a critical effect on the original system by changing such a combination. A pairwise mapping to *θ*_25/*wee *_∈ **p***^hyb ^*can be achieved by simultaneous changes in *V*_*i*25 _∈ **p***^org ^*and *V_awee _*∈ **p***^org ^*or *V*_*a*25 _∈ **p***^org ^*and *V*_*iwee *_∈ **p***^org^*. By adding the parameter *s *∈ **p***^org ^*such that *V*_*i*25 _→ *sV*_*i*25_, *V_awee _*→ *sV_awee_*, and the parameter *t *∈ **p***^org ^*such that *V*_*a*25 _→ *tV*_*a*25 _and *V*_*iwee *_→ *tV_iwee_*, then a perturbation of *s *∈ **p***^org ^*or *t *∈ **p***^org ^*in the original model corresponds to changing *θ*_25/*wee *_∈ **p***^hyb^*. Using the same perturbation scheme as before (wide range) we obtained the following sensitivity scores for the combined perturbations  and , which can be compared to the score of *S^osc ^*= 0 for *V*_*i*25_, *V_awee_*, *V*_*a*25 _and *V*_*iwee*_, respectively. This shows that by combining perturbations of two, by themselves, robust parameters, we can obtain a critical effect.

#### Modulatory combinations of parameters

From the hybrid model we retrieved an expression for the critical cell mass, *M_C_*, above which mitosis is initiated [15](1)

where all constants are hybrid model parameters. Mathematically, this corresponds to a bifurcation point at which the system moves from a stable fixed point to a limit cycle. Under circumstances corresponding to a normal cell, this expression can be used to approximate final cell mass before cell division.

If we assume that , *l*_25 _<<*h_wee_*, as for the normal cell (equation (20)), then equation (1) can be approximated with(2)

Using this expression, we can compute quantitative predictions of cell size, e.g. if *k*_1 _is reduced by 50%, the cell size is expected to be twice as large. This was tested in the original model by setting *k*_1 _= 0.015 and running a numerical simulation. Final cell mass increased from 2.0 to 4.0. If, together with halving *k*_1 _we also double *θ*_25/wee _and *h_wee_*, cell size should increase by about 2 × 2 × 2 = 8 times. Testing this in the original model by setting *k*_1 _= 0.015, *V*_*i*25 _= *V_awee _*= 0.5 (corresponding to doubling the hybrid model parameter θ_25/wee _),  (doubling *h_wee_*), final cell size increased from 2.0 to 14.4. We can therefore predict multiplicative effects by using the hybrid model.

### Summary of results

In this case study of the cell cycle, by comparing the sensitivity analysis of the original model with analytical predictions from the hybrid model, we found that i) predictions for essential and modulatory parameters from the hybrid model were satisfactory for small perturbations, but more discrepancies were seen for larger perturbations; ii) small perturbations were of more use when trying to identify important modulatory mechanisms compared to larger ones whereas, iii) larger perturbations were needed in order to identify essential parameters. Furthermore, iv) most parameters were either essential or modulatory using this set of perturbations. Finally, v) the most sensitive feature of the system (the threshold corresponding to *θ*_25/*wee*_) was robust towards single parameter perturbations.

## Discussion and Conclusions

Any biological process is essentially an interaction between different elements in time and space. Given experimental data and prior knowledge we may be able to formulate such interactions in mathematical terms. As a rule, such models suffer from uncertainty in the structure, both from a lack of representation of essential elements and interactions, as well as from erroneous model representation of the phenomena under investigation [21,22]. Moreover, parameters are uncertain in the sense that several different combinations are consistent with a given set of data. When dealing with a specific model, such as the NT-model for the cell-cycle [19], we are in a unique position because a large body of experimental work has already been encoded in mathematical models by the pioneering work of Novak and Tyson [23]. Furthermore, this specific NT-model has been analyzed using both bifurcation [19] and mathematical piecewise linear approximation techniques [15]. Bifurcation analysis indicates the dynamical mechanisms behind the transitions within the cell-cycle, while the mathematical simplification analysis provides proof of the existence of a limit cycle, yields explicit analytical expressions describing how model parameters affect cell size, and also provides conditions necessary for oscillations. However, it is generally very difficult to succeed with a thorough mathematical analysis of a given computational model, and this becomes even harder for larger models. Numerical bifurcation analysis is also a technique that is most efficient for smaller models. For larger models, we are left performing numerical simulations to explore the sensitivity of the model, and thereby increase our understanding of what parts of the model may be important for different aspects of the system's behaviour [24]. For these reasons, we have here interpolated between the results obtained from a sensitivity analysis of the original model, and the outcome from a previous model simplification [15], with the rational being that such an analysis may be instructive for assessing what we can and cannot discover using a sensitivity analysis. Such understanding may prove useful for those cases where an explicit mathematical simplification is not feasible.

Our analysis of the simplified model resulted in the formulation of two types of mathematical constraints - one identifying parameter perturbations with a qualitative effect on model output (NC), and the other describing perturbations with a quantitative effect (SF). From the corresponding sensitivity analysis of the original NT-model, we could also here identify perturbations that had a qualitative effect (removing oscillations), or quantitative effect (changing cell size). Using the same set of parameter perturbations and sensitivity measures in both the hybrid and original models, we could compare our analytical predictions to the sensitivity analysis. Based on our definition on modulatory and essential parameters, both methods characterized the parameters more or less equally for narrow range perturbations; for wide range perturbations there was a discrepancy.

This combined analysis demonstrated that the parameters were, in most cases, characterized as either essential or modulatory using this set of perturbations, where essential parameters were only identified using wide perturbations. This means that there are parameters that do not have any effect at small perturbations, not even modulatory, but which do have a critical (essential) effect at large perturbations. They are therefore very important to the system even though this would not be obvious from a local sensitivity analysis. Modulatory parameters, as defined by the size function (SF) of the hybrid model, were best identified by small perturbations. When larger perturbations were applied, parameters from other parts of the system also showed up, confusing the picture. SF describes the parameter relationship of the G2/M checkpoint - the proteins behind this checkpoint are those that are most important in cell size regulation. If we did not know this and were to try to identify the mechanism regulating cell size, we would be better to use small perturbations. Essential parameters, on the other hand, could not be found using small perturbations alone; rather larger perturbations had to be used. This is not surprising considering that the biological system has to be robust in the default parameter setting, and the corresponding point in parameter space must therefore be located far away from the region where there are, for example, no oscillations. Using the hybrid model for more complex predictions and comparing these to the sensitivity analysis, we found that the threshold corresponding to the hybrid parameter *θ*_25/*wee*_, which has an essential effect (and modulatory) in the hybrid model, were robust (using *S^osc^*) towards single parameter perturbations; however it had an essential effect with a targeted combined perturbation. We also demonstrated the possibility to find combinations of parameters with a multiplicative effect on cell size.

In the case of single parameter perturbations, it is possible to numerically simulate the behaviour of a large model to all possible parameter perturbations. With perturbations of more than one parameter at a time, the combinatorial explosion has the effect that all parameter combinations cannot be tested. A simplified model therefore has the potential to inform us about which parameter combinations that are important to test. Our study pin-points the difference between parameters that have a quantitative, modulatory impact on model output, where the perturbation size is correlated with the change in model output, and parameters that are essential to the system in a qualitative manner.

Our investigation has several similarities to a recent study of Radulescu et al [14] where they suggest the use of a hierarchy of reduced models to describe complex biological networks. Radulescu also map critical parameters identified from the reduced model, back to the original model. However, there are also marked differences. In Radulescu et al, model reductions are by and large faithful in both structure and components to the original model, letting kinetics guide structural decomposition and parameter elimination, keeping the most important components and not introducing new ones. In the process, critical monomials and thereby critical parameters are identified. This is not the approach taken here. There are no simple and direct analytical relationships between the original and simplified models. Instead we look at different perturbations in the original model and recalibrate the simplified model from those in order to backtrack findings in the simplified model. By using a new model formalism, in this case, a piecewise linear with delay, we can point to other important features of the original model. In this case that the system seems to switch between different linear systems and that the location of the fixed points and the thresholds are most important for the dynamics. We also find that a very sensitive feature of the original model, the threshold described earlier, is robust towards single parameter perturbations.

A simplified model is an approximation of a more complicated system, and there will be instances when it does not work. Its predictions must therefore be confirmed in the full model context. However, a discrepancy between the simplified and original model is of interest in itself, since it can point out an approximation that has failed. Since approximations are a way to learn about the essentials of a system, this gives information on neglected essential features. To combine model simplification with sensitivity analysis is a way to investigate the structure of complex biological networks. We believe that this approach is applicable to systems other than the cell cycle, even though the precise set up used in this study may not be suitable. Sensitivity analysis has to be carefully performed, since it is almost impossible to investigate the full parameter space for any model with more than a few parameters. Here we performed a first step screening study, modifying one parameter at a time. The next logical step would be to consider a more global sensitivity analysis by investigating combinations of parameters, which was beyond the scope of this study. In this comparison between the hybrid model and the original model, we have considered all simulations with a quantized behaviour as not working properly, and therefore disqualified them from the analysis. This is partly because a normal cell does not display this type of behaviour, but also because the hybrid model does not mimic the behaviour of the original model in a few of these quantized instances.

## Methods

### The original NT-model

The original Novak and Tyson model [19] which was our point of departure corresponds to(3)(4)(5)(6)(7)(8)(9)(10)(11)(12)(13)(14)

where(15)

The default parmater values corresponding to the wild-type cell are(16)

### The hybrid, DPL-model

The hybrid model approximation process is described in [15] and summarized further down. Let  represent the state of the cell cycle system ( corresponds to NT-model variable [Cdc13_T_] and *x*_PreMPF _to NT-variable [preMPF]). Further, let *u_ext_*(*t*) = *M *(*t*) (cell mass) be the external input, and *y*(*t*) the output from the system (DPL-model variable *y *corresponds to NT-variable [MPF] and we sometimes, a bit sloppy, write *y *= [MPF]). Then, the DPL-model can be written as(17)

where **A***_kl _*is a 2 × 2 matrix, **B **= (*k*_1 _0)', **C **= (1 -1), *k*_1 _a constant parameter, and the following rules are used for switching between linear systems(18)

Here *θ*_25/*wee*_, *θ*_*slp*/*ste *_and *τ *are constant parameters retrieved from the model approximation process. The variable *u_ext_*(*t*) = *M *(*t*) is most often treated as a constant parameter *u_ext _*= *M*, otherwise it evolves according to . From the approximation process, the system matrices will correspond to(19)

where , *l_slp/ste_*, *h_wee_*, *l*_25_, *h_slp/ste_*, *l*_wee _and *h*_25 _are constant parameters. In the wild-type cell the use of **A**_11 _corresponds to cell cycle phase S/G2, **A**_12 _to phase G1, **A**_21 _to Mitosis and **A**_22 _to ending of Mitosis. The default parameter values used are(20)

This default parameter set was retrieved by calibrating the hybrid model to the unperturbed original NT-model, as described below. It differs slightly from the default parameter set of [15].

### Parameter perturbations

Each simulation was run in 4000 minutes using the numerical integration program XPP and integration method QualRk. We defined one cell division cycle as the trajectory between two local minima in the mass *M *variable. The length of each such cycle, the cycle time, *t_CT_*, was recorded. If the trajectory still oscillated after 4000 minutes and the distribution of *t_CT _*was narrow (within 1 min from average *t_CT _*), the perturbation was defined as having proper oscillations.

### Size Function

In [19] and [15] the dynamics of the cell cycle are described in terms of bifurcation analysis, presuming that the size of the cell (cell mass *M*) changes much slower than the other variables and therefore can be viewed as a constant parameter in the analysis of the dynamics. From a bifurcation diagram (e.g. Figure six in [15]) using *M *as bifurcation parameter and [MPF] as bifurcation variable, can be found that, during normal circumstances, critical for the size of the cell is the point of bifurcation when the system moves from a stable fixed point to a limit cycle. In [15] we gave an analytical expression for when this bifurcation takes place.

In the coming discussion we use the notation of the hybrid (DPL) model (equations (17-19)), and view *M *as a constant parameter. Each linear system of the DPL-model (defined by the system matrix *A_kl_*), has a fixed point . The location of  in the phase space of **x **thus depends on *M*, i.e. . The bifurcation described above takes place at the critical cell mass, *M_C_*, at which  of the linear system defined by *A*_11_, is located on the switching threshold corresponding to *θ*_25/wee_, i.e. , which, using the notation of equations (17-19) gives the following expression for the critical cell mass *M_C_*,(21)

At this point the system switches from the dynamics of a stable fixed point to a limit cycle. After entering the limit cycle the cell will divide approximately *τ *minutes later, corresponding to a final cell mass of(22)

Presuming that for small perturbations this relationship will remain, we use equation (22) to predict the effect of perturbations in the hybrid and (after translation) the original model.

### Necessary conditions

In the analysis of the hybrid (DPL) model [15] we retrieved two conditions, necessary in order to have cell cycle oscillations, namely

and

where we use the notation of equations (17-19),  is the slow eigenvector of *A*_12_, going through the fixed point  and *M_end _*is given from equation (22). Note that *M_end _*was calculated from *M_C_*, i.e. the point where **Cx**_11 _passes *θ*_25/*wee *_as described above.

For larger perturbations affecting *θ*_*slp*/*ste *_another fixed point and switching threshold relationship can be decisive for cell size, namely **Cx**_21 _passing *θ*_*slp*/*ste *_and final cell size can therefore be extended to(23)

were  and the conditions therefore corresponds to(24)

and(25)

which are used as necessary conditions throughout this study.

### Summary of the model simplification process

The model simplification process [15] included the following steps; where **p***^org ^*is the set of original parameters and **p***^hyb ^*the set of hybrid (DPL) model parameters.

1. The steady-state input/output behaviour of subsets of variables of the original model were graphed.

2. Variables with minor effect on the dynamical behaviour were removed.

3. Remaining variables were lumped into switching modules *SM_s _s *∈ 1,..., 3, such that one variable was functioning as input to the module, a combination of variables as output, and the steady-state input/output behaviour were in the form of a monotonic function with a sigmoid kind of shape. The input/output function is denoted, , where  is a subset of original model parameters; those parameters that exist in the ODE equations defining *SM_s_*.

4. The sigmoid functions  were approximated by step functions , where , and *θ_s _*corresponds to the switching threshold of the step function, *h_s _*(high) to the maximum level and *l_s _*(low) to the minimum level (*θ_s_*, *h_s_*, and *l_s _*are in this study determined from three points of  as described in the calibration below).

5. The switching modules *SM_s _*were each approximated by a step function  and a time delay *τ_s _*[15]. The time delay was often *τ_s _*= 0.

Note that we here only consider the small DPL-model in [15].

### Mapping (calibration) procedure

In order to construct mappings between **p***^org ^*and **p***^hyb^*, all parameters of **p***^org ^*were perturbed *n_pert _*= 11 times according to **ps **(defined in Results), and for each perturbation a calibration of the hybrid model was performed. The hybrid model corresponding to the *j*th, *j *∈ 1,...,11 perturbation of parameter  is denoted .

The calibration of the step functions  was done from three points of the sigmoid functions , *s *∈ 1,...,3, corresponding to the threshold, the high, and low level of the step-functions. The threshold level was defined as the point (denoted *θ^SM^*) where the derivative of the curve  was the largest (smallest), and the high and low level were measured at two points (denoted *h^SM ^*and *l^SM^*) of the curve  corresponding to  or , depending on whether the sigmoid was increasing or decreasing;  corresponds to *θ^SM ^*using the default parameter set. The hybrid model calibration procedure for mapping between original parameter perturbations and hybrid parameters can be described in pseudo-code as

   **for **each , *i *∈ 1,..., *n_org _***do**

      **for **each perturbation size *ps_j _*∈ **ps**, *j *∈ 1,..., *n_pert _***do**

         set ;

         let  be formed according to:

         **for **each switching module *SM_s _s *∈ 1,..., 3 **do**

            measure  and ,  of ;

            use  and ,  as values for parameters *θ_s_*, *h_s_*, ;

         **end for**

      **end for**

   **end for**.

The hybrid parameter *τ *were not included in the comparison between the two models of practical reasons; since this parameter can not easily be mapped via  as the other ones. From this process we retrieved *n_org _*× *n_pert _*= 47 × 11 sets of hybrid model parameters , *j *∈ 1,..., *n_pert_*.

To translate the notation above to the hybrid model parameters of equation (20) let *s *∈ 1,..., 3 = {25, *wee*, *slp*/*ste*}, except for *θ*_25 _and *θ_wee_*. During the model simplification process we noted that  (when using default parameters) and this was used as a further simplification step by merging *θ*_25 _and *θ_wee _*into one parameter θ_25/*wee*_. Therefore, only perturbations of the original model which keep the  property are consistent with the hybrid model. These are all pairwise perturbations.

## Authors' contributions

OE, TA and JT conceived of and designed the study and drafted the manuscript. OE performed the analysis. YZ gave central contributions to the analysis of the hybrid model. All authors read and approved the final manuscript.
